# Pain Management in Older Adults: An Interprofessional Training Approach Assessed by Script Concordance Tests

**DOI:** 10.1111/papr.70208

**Published:** 2026-10-03

**Authors:** Alexandre Meurant, Vincent Bonnet, Clément Gakuba, Cédric Villain, Gilles Loggia

**Affiliations:** ^1^ Department of Geriatric Medicine Normandie Univ, UNICAEN, CHU de Caen Caen France; ^2^ Department of Pharmacy Normandie Univ, UNICAEN, CHU de Caen Caen France; ^3^ Department of Anesthesiology and Critical Care Polyclinique du Parc Caen France; ^4^ Department of Anesthesiology and Critical Care Normandie Univ, UNICAEN, CHU de Caen Caen France; ^5^ Normandie Univ, UNICAEN INSERM, PhIND, CYCERON Caen France; ^6^ Normandie Univ, UNICAEN INSERM, COMETE, CYCERON Caen France; ^7^ Post‐Acute Care and Rehabilitation Department Normandie Univ, UNICAEN, CHU de Caen Caen France

**Keywords:** adverse effect management, analgesic prescribing, elderly patients, interprofessional education, pain assessment, script concordance test

## Abstract

**Background:**

Optimal perioperative pain management in older adults challenges anesthesiologists due to increased drug sensitivity, altered pharmacokinetics, and limitations of pain assessment tools in cognitively impaired patients. This study evaluated whether a single 60‐min structured collaborative training session between a geriatrician and a clinical pharmacist is associated with improved anesthesiologists' clinical reasoning regarding perioperative analgesic management in older adults, as measured by increased concordance with expert opinion on a script concordance test (SCT).

**Methods:**

SCT assessed 20 clinical vignettes covering pain assessment, analgesic selection, and adverse effect management domains. These were submitted to anesthesiologists before and after training. A panel of 13 geriatricians established scoring criteria. Internal consistency was measured using Cronbach's alpha. Paired *t*‐tests and Wilcoxon signed‐rank tests compared pre‐ and post‐training scores, with the 95% confidence interval (CI) for the overall change estimated by bootstrap resampling.

**Results:**

Seventeen anesthesiologists completed both assessments. Mean SCT scores increased from 45.8 ± 7.4 to 59.5 ± 8.5 post‐training (*p* < 0.001), showing a 29.7% improvement (95% CI, 18.7%–41.7%). Analysis revealed improvements in analgesic prescription (+47.9%; 95% CI, 10.0–28.1 points) and adverse effect management (+26.8%; 95% CI, 9.1–19.0 points), while the change in pain assessment was not statistically significant (−11.6%; 95% CI, −16.6–9.4 points). Internal consistency improved from 0.542 to 0.704. Most participants (89.5%) lacked prior training in prescribing analgesics to older adults, and 73.7% were unfamiliar with clinical guidelines.

**Conclusion:**

This study suggests that collaborative geriatrician‐pharmacist training is associated with improved anesthesiologists' clinical reasoning regarding perioperative analgesic management in older adults, as measured by greater concordance with expert opinion on a script concordance test. The varying effects across domains suggest that distinct competencies may require distinct educational approaches. These findings support further investigation of interprofessional education into anesthesiology training to enhance clinical reasoning in the care of older surgical patients.

## Key Points

- Interprofessional geriatric‐pharmacist training was associated with improved anesthesiologists' clinical reasoning in geriatric pain management (SCT +29.7%).
- SCT scores increased 29.7% (95% CI, 18.7%–41.7%) after collaborative educational intervention.
- Analgesic prescription scores improved 47.9%; adverse effects 26.8%.
- Junior anesthesiologists showed greater improvement than senior colleagues.
- Interprofessional education shows promise for improving clinical reasoning in geriatric perioperative care.

## 1 Introduction

Perioperative management of older adults is challenging for anesthesiologists. With an aging population and more surgeries in older adults, optimal pain management is key to Enhanced Recovery After Surgery (ERAS) protocols, which promote multimodal analgesia and reduce opioid use [1].

Age‐related physiological changes, including reduced renal and hepatic functions, altered body composition, and lower plasma protein levels, affect analgesic pharmacokinetics. These changes lead to drug accumulation and increased plasma concentrations. Pharmacodynamics are modified by increased brain sensitivity to psychotropic drugs and anticholinergic effects. Multimorbidities and polypharmacy complicate anesthetic management through drug interactions and increase the adverse effects [2].

Pain management in older adults requires specific considerations, owing to altered pharmacokinetics, increased medication sensitivity, and a higher risk of adverse reactions. Comorbidities complicate management and require caution with common analgesics [3]. Nefopam is potentially inappropriate because of its anticholinergic properties. NSAIDs are contraindicated in patients with renal or heart failure. Opioids require laxative co‐prescription to prevent constipation, and recommendations suggest a 25%–50% dose reduction in older patients following the “start low, go slow” principle [4].

ERAS protocols have shown significant benefits for older surgical patients, reduced hospital stays, and improved recovery [5]. Analgesic prescriptions for older adults require an understanding of the medication risks and patient needs.

Script Concordance Testing (SCT) evaluates clinical reasoning under uncertainty using scenarios that reflect real clinical situations [6]. SCT effectively evaluates reasoning in geriatric pain management by assessing decision‐making in complex scenarios in which multiple strategies may be applied. This is a significant challenge for anesthesiologists in managing older adults with comorbidities and physiological changes. Well‐validated in geriatric medicine [7] and anesthesiology [8, 9], SCT helps assess the quality of medical decision making and identify areas requiring improvement. Despite the importance of specialized geriatric pain management knowledge among anesthesiologists, there remains a gap in targeted educational interventions. While studies have examined SCT in anesthesiology [8, 9] and geriatric medicine [7], research on the interprofessional training of anesthesiologists in geriatric pain management is limited.

We hypothesized that a collaborative educational intervention by geriatricians and pharmacists would improve anesthesiologists' clinical reasoning regarding perioperative analgesic prescriptions for older patients, as measured by SCT scores. Our objective was to evaluate whether this training improved anesthesiologists' clinical reasoning, assessed as concordance with an expert panel, regarding analgesic management in older adults.

## 2 Methods

### 2.1 Study Design and Participants

We conducted a prospective pre‐post intervention study at a private medical‐surgical‐obstetrical hospital (250 beds) in Caen, Normandy, France between May and October 2023. The study comprised three phases: initial assessment (1 month), educational intervention (1 month), and post‐intervention assessment (1 month), with follow‐up periods between phases.

All practising anesthesiologists (*n* = 19) were invited to participate in the study. The inclusion criteria were active practice at the study site, regular prescription of analgesics for older patients (≥ 65 years) in perioperative settings, and willingness to complete both assessments. No exclusion criteria were applied to maximize ecological validity. Participation was voluntary and written informed consent was obtained from all participants according to the institutional protocol.

The sample size determination was pragmatic and included all eligible anesthesiologists to achieve maximum participation. This approach prioritizes ecological validity over statistical power calculations, which is consistent with pragmatic educational research design. With 90% participation, we estimated 17–18 participants would complete the study. Previous SCT‐based studies with similar sample sizes [8] have detected meaningful training effects.

The SCT instrument comprised 20 clinical vignettes across three domains based on SCT guidelines [10, 11] and expert consensus: analgesic prescription (*n* = 7), adverse effect management (*n* = 11), and pain assessment (*n* = 2). The distribution reflected each domain's importance in perioperative geriatric care, with adverse effect management receiving the most items, given its role as a prescription barrier (confirmed by 94.4% of participants citing this as a major concern). The limited pain assessment vignettes represent a pragmatic decision based on available expert‐validated scenarios, although this may limit statistical power in this domain.

### 2.2 Development and Scoring of the Script Concordance Test

The SCT was developed according to the guidelines of the University of Montreal and the French National Authority for Health [10, 11]. The test involved the selection of clinical scenarios, including pain assessment, analgesic selection, and adverse effect management. Candidate vignettes were drafted by the geriatrician‐pharmacist team from real perioperative situations encountered in older adults and from current analgesia guidelines, and were reviewed for clarity, plausibility, and clinical relevance by an academic geriatrician. Items were revised or discarded on this basis before finalization of the instrument. The final set of items was then independently answered by the panel of 13 geriatricians, who were not involved in the construction, review, or selection of the vignettes; the distribution of their responses on the −2 to +2 scale defined the scoring key. Scores therefore reflect concordance with aggregated expert opinion rather than with a single external reference standard. The complete SCT instrument is provided in Supporting Information S1.

Each SCT item comprised (1) a clinical vignette describing an uncertain situation, (2) a proposed diagnostic or therapeutic option, and (3) new information. Participants evaluated how this new information affected the initial option using a five‐point Likert scale (−2 to +2), indicating whether the option became more justified, unchanged, or less justified. As shown in Figure S1, this format assesses clinical decision‐making under uncertainty by scoring based on expert opinions.

Participants rated whether the new information made the initial approach more justified (+1, +2), unchanged (0), or less justified (−1, −2). The items were independent and assessed clinical reasoning rather than factual recall. The instrument was reviewed by the geriatrician–pharmacist team and an academic geriatrician, with refinement using the Montreal Centre for Applied Pedagogy quality control matrix.

Scoring followed Norman and Norcini's aggregate method, acknowledging the variations in expert clinical judgment. The algorithm assigned full credit (1.0) to modal expert responses, with partial credit proportional to expert frequency, as shown in Figure S1. The non‐expert responses received no credit. Scores were summed and transformed into a 100‐point scale [12].

Following the established SCT methodology [12], we used identical tests for pre‐ and post‐training assessments. While this enables direct comparison, it may introduce test familiarity effects, which are addressed in the limitations section.

### 2.3 Data Collection

Data were collected in three phases: baseline assessment (participants completed questionnaires on demographics, clinical experience, prescribing practices, and geriatric pain management guidelines, followed by baseline SCT), educational intervention (participants attended training), and post‐intervention assessment (1 month later, participants completed an identical SCT). All assessments were conducted electronically using SurveyMonkey, with anonymized identifiers.

### 2.4 Educational Intervention

The training involved in‐person sessions with anesthesiologists and digital materials (Supporting Information S2). A geriatrician‐pharmacist team presented a 60‐min session on pain assessment in older patients, analgesic treatments (acetaminophen, NSAIDs, nefopam, opioids, and neuropathic pain), clinical algorithms, postoperative pain protocols, and drug precautions. It emphasized “start low, go slow” principles, opioid adverse effect prevention, and pain reassessment in cancer patients. Subsequently, the participants discussed the clinical cases with specialists in a Q&A session.

### 2.5 Statistical Analysis

Descriptive analyses were performed on all data. Score distributions were tested for normality using the Shapiro–Wilk test. Internal consistency was evaluated using Cronbach's alpha. Paired Student's *t*‐tests were used to compare pre‐ and post‐training mean scores as they analyzed repeated measurements before and after intervention, controlling for individual variations. Wilcoxon signed‐rank tests were also used given the small sample size. The change in the overall score is reported as a mean paired difference with a 95% confidence interval (CI); the 95% CI of the percentage change was obtained by bootstrap resampling of participants (10,000 resamples). A two‐sided *p* < 0.05 was considered significant. Complete data were available for all participants who completed both assessments (*N* = 17). No imputation methods were required because the analysis was restricted to participants with complete data. Analyses were performed using IBM SPSS software (version 26.0).

Based on previous SCT studies [9], we pre‐specified that a 6% difference between pre‐ and post‐training scores would represent a meaningful improvement in clinical reasoning.

## 3 Results

### 3.1 Participant Characteristics

As shown in Figure S2, 19 anesthesiologists participated in the baseline assessment, of which 17 (89.5%) completed both the pre‐ and post‐training evaluations. Two participants (10.5%) did not complete the post‐training assessment despite multiple reminders. None of the participants withdrew their consent. All participants who completed both assessments provided complete data for all the SCT items. Table 1 presents the participant characteristics and baseline practice patterns. Most participants (89.5%) had never received dedicated training in analgesic prescription for older patients, although all reported regular treatment for older adults.

**TABLE 1:** Assessment of practices in the study population (*n* = 19).

Characteristic	Anesthesiologists *n* = 19
Experience as an analgesic prescriber	
6 to 10 years	6 (31.6)
11 to 20 years	8 (42.1)
More than 20 years	5 (26.3)
Number of elderly patients treated per month	
Between 11 to 50	11 (57.9)
Between 51 to 100	5 (26.3)
More than 100	3 (15.8)
Familiarity with guidelines on pain management in older adults	5 (26.3)
Previous training on analgesic prescription in older adults	2 (10.5)
Types of analgesics most frequently prescribed	
Acetaminophen	19 (100)
Non‐steroidal anti‐inflammatory drugs	7 (38.9)
Level II (weak) opioid analgesics	14 (77.8)
Level III (strong) opioid analgesics	10 (55.6)
Other	4 (22.2)
Obstacles encountered in prescribing analgesics	
Drug interactions	8 (44.4)
Potential adverse effects	17 (94.4)
Difficulties in pain assessment	8 (44.4)
Patient preference	2 (11.1)
Inability to relieve the patient	2 (11.1)
Use of specific tools for pain assessment in older adults	2 (10.5)
Assessment of current pain management in facility	
Insufficient	1 (5.3)
Average	11 (57.9)
Good	7 (36.8)

*Note:* Values are number of subjects (percentage).

Acetaminophen was universally prescribed (100%), followed by weak (77.8%), strong opioids (55.6%), and NSAIDs (38.9%). The most frequently cited barriers to optimal prescription were concerns about adverse effects (94.4%), drug interactions (44.4%), and difficulties in pain assessment (44.4%). Only 10.5% of participants used validated pain assessment tools for older adults.

### 3.2 Impact of Training on SCT Scores

As illustrated in Figure 1A, the mean scores increased from 45.8 ± 7.4/100 pre‐training to 59.5 ± 8.5/100 post‐training (*p* < 0.001), representing a 29.7% improvement (mean paired difference, +13.6 points; 95% CI, 8.5–18.8; 95% CI of the relative change, 18.7%–41.7%). Cronbach's α changed from 0.542 to 0.704 after training. The post‐training value reached the threshold for acceptable internal consistency (*α* ≥ 0.70), although the interpretation of this improvement is complex, given identical test administration and the absence of comparative normative data for changes in internal consistency following educational interventions.

**FIGURE 1:**
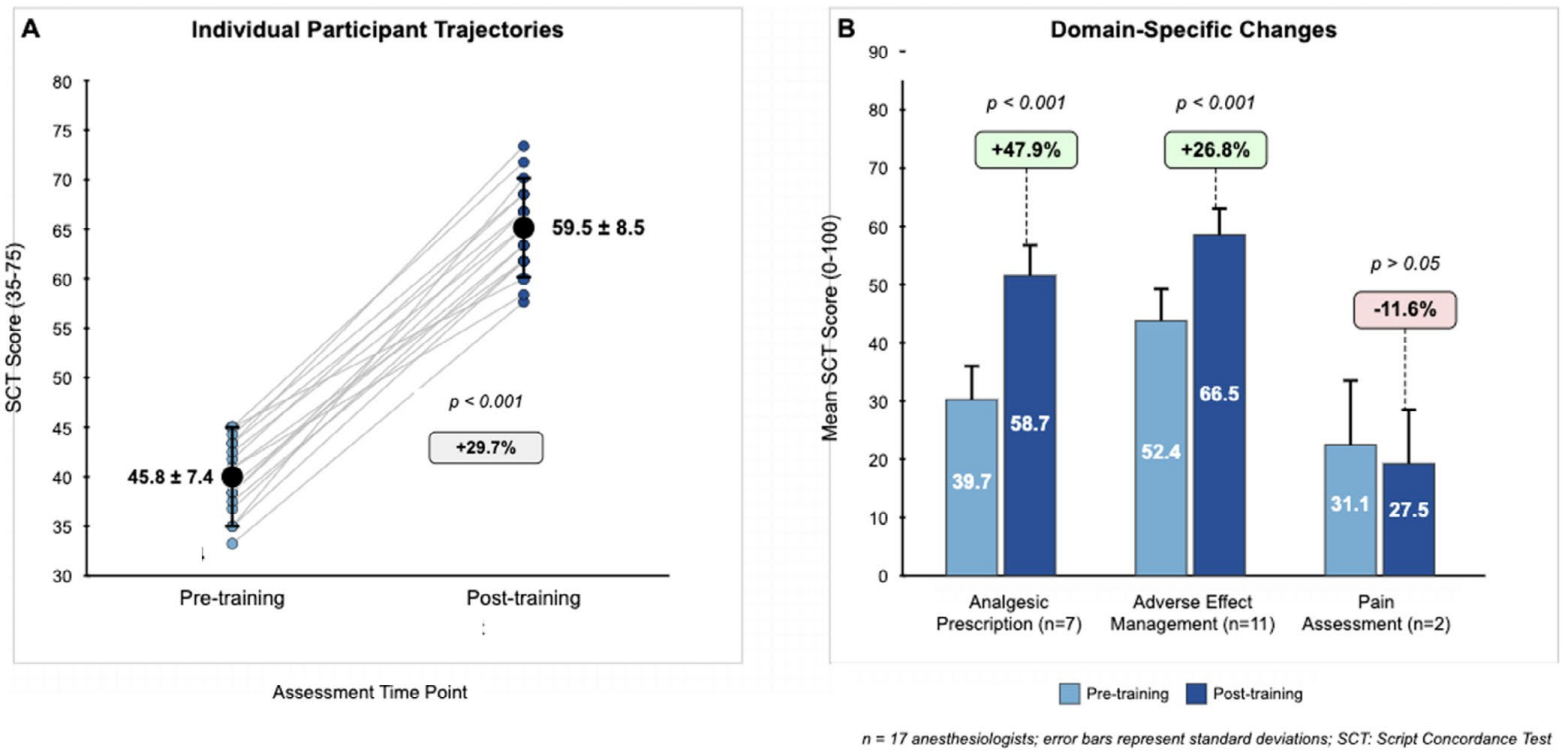
Changes in script concordance test scores following collaborative training. Panel (A) displays individual participant trajectories (*n* = 17) with pre‐training (light blue circles) and post‐training (dark blue circles) SCT scores. Means ± standard deviations are shown with black circles and error bars (pre‐training: 45.8 ± 7.4; post‐training: 59.5 ± 8.5; *p* < 0.001; +29.7% improvement; 95% CI, 18.7%–41.7%). Panel (B) shows domain‐specific changes in the mean SCT scores across three clinical domains: Analgesic Prescription (*n* = 7), Adverse Effect Management (*n* = 11), and Pain Assessment (*n* = 2). Statistically significant improvements were observed in Analgesic Prescription (+47.9%, *p* < 0.001; 95% CI, 10.0–28.1 points) and Adverse Effect Management (+26.8%, *p* < 0.001; 95% CI, 9.1–19.0 points), while Pain Assessment showed a non‐significant change (−11.6%, *p* > 0.05; 95% CI, −16.6–9.4 points). Error bars represent standard deviation.

### 3.3 Domain‐Specific Changes

Domain‐specific analysis (Figure 1B) revealed differential changes in key areas. The most substantial improvement occurred in analgesic prescription, with scores increasing from 39.71 ± 11.38/100 to 58.74 ± 12.51/100 (*p* < 0.001; mean paired difference, +19.0 points; 95% CI, 10.0–28.1), a 47.9% improvement in the post‐intervention phase. For adverse effect management, scores improved from 52.42 ± 10.45/100 to 66.48 ± 9.92/100 (*p* < 0.001; mean paired difference, +14.1 points; 95% CI, 9.1–19.0), a 26.8% increase in mean score.

In the pain assessment domain, which included only two items, scores changed from 31.09 ± 17.78/100 pre‐training to 27.50 ± 16.54/100 post‐training, representing a non‐significant change of −11.6% (*p* > 0.05; mean paired difference, −3.6 points; 95% CI, −16.6–9.4).

### 3.4 Experience‐Based Differences

Further analysis revealed different training effects based on the clinical experience (Table 2). The most pronounced improvements occurred among junior‐ and mid‐career anesthesiologists, whereas those with more than 20 years of experience showed no statistically significant changes.

**TABLE 2:** Impact of training by years of clinical experience.

Experience level	Pre‐training score	Post‐training score	Change (%)	*p*
6–10 years (*n* = 4)	43.2 ± 6.5	61.7 ± 7.3	+42.8	0.001
11–20 years (*n* = 8)	46.5 ± 7.9	60.2 ± 9.1	+29.5	0.004
> 20 years (*n* = 5)	48.3 ± 6.1	54.7 ± 7.4	+13.3	0.09

*Note:* Values are mean ± SD.

### 3.5 Participant Feedback

Qualitative feedback indicated high satisfaction with the training format and the program content. Participants valued their interactions with specialists and intended to implement the recommendations in clinical practice, particularly regarding analgesic selection and prevention of adverse effects.

## 4 Discussion

Our study suggests that collaborative training between a geriatrician and a clinical pharmacist is associated with improved performance on a script concordance test, reflecting greater concordance with the aggregated opinion of an expert geriatric panel regarding perioperative analgesic management in older adults.

### 4.1 Interpretation of Primary Findings

The significant improvement in overall scores (29.7%; 95% CI, 18.7%–41.7%) following the educational intervention exceeded the pre‐established threshold (6%). This aligns with Compère et al. [13], who demonstrated the effectiveness of tutored practice exchange groups in improving clinical reasoning among anesthesiology residents. This improvement is noteworthy given the complexity of geriatric pain management and the short training program duration.

The enhanced internal consistency (Cronbach's alpha improvement from 0.542 to 0.704) indicated more coherent clinical reasoning patterns among participants. This transition to acceptable reliability shows the development of consistent frameworks for geriatric pain management decisions, in which practice standardization improves postoperative outcomes [14]. As Lubarsky et al. [6] noted, improvements in internal consistency reflect the maturation of clinical reasoning after an educational intervention.

### 4.2 Domain‐Specific Effects: Implications for Anesthetic Practice

The domain‐specific analysis revealed differential effects across the three competencies, offering insights into possible educational priorities.

The observed improvement in analgesic prescription scores (+47.9%, *p* < 0.001) indicates enhanced clinical reasoning regarding analgesic selection in older adults. Lan et al. [15] demonstrated that appropriate analgesic selection in older patients reduced postoperative delirium. Given the association between suboptimal analgesic selection and increased complications and hospital stay [16], improved reasoning in this domain may be clinically relevant, although our design does not allow conclusions about prescribing behavior or patient outcomes.

The improvement in adverse effect management (+26.8%, *p* < 0.001) was relevant, as 94.4% of participants identified adverse effects as a major prescribing barrier. This domain requires integration of pharmacological knowledge with patient‐specific factors. The more limited gain relative to the prescription domain may reflect the complexity of anticipating medication effects in heterogeneous older populations [17]. Studies have shown that older patients are vulnerable to adverse analgesic effects, including respiratory depression, cognitive dysfunction, and acute kidney injury [18].

The change in pain‐assessment scores was not statistically significant (−11.6%, *p* > 0.05). Because this domain comprised only two items, the analysis might have been underpowered, and no conclusion can be drawn regarding the effect of training on pain‐assessment reasoning.

### 4.3 Experience‐Based Differences in Educational Impact

Clinical experience influenced the training impact, with junior and mid‐career anesthesiologists showing greater improvements than their senior colleagues. This aligns with the literature in which Martinelli et al. [19] found that early‐ and mid‐career practitioners demonstrate higher receptivity to innovative methods and technologies.

Anesthesiologists with 6–10 years of experience showed a strong improvement (+42.8%, *p* = 0.001), suggesting an optimal window for educational interventions in which practitioners can contextualize new information while maintaining flexibility. Midcareer anesthesiologists often serve as clinical leaders, potentially amplifying their impact through peer influence [20].

Among those with > 20 years of experience the change was not statistically significant (+13.3%, *p* = 0.09), most likely reflecting the limited power of this small subgroup (*n* = 5); this finding is therefore inconclusive. The higher baseline competency in this group is consistent with reports on the challenges of balancing established and newer approaches [19, 21].

### 4.4 Implications for Anesthesiology Practice and Training

This study's findings have several practical implications. First, the observed improvement in clinical reasoning regarding analgesic selection suggests that integrating geriatric pharmacology and pain management principles into anesthesiology training and perioperative protocols deserves consideration. Such integration could translate into the development of age and comorbidity‐stratified analgesic protocols with dosage adjustments based on renal and hepatic function, as recommended by the American Society of Anesthesiologists [22].

Second, the results highlight the importance of inter‐professional collaboration in perioperative care. Regular interactions with geriatricians and clinical pharmacists may help anesthesiologists in complex cases, aligning with models such as preoperative optimization services that demonstrate benefits for older adults [23]. Integrating clinical pharmacists into perioperative teams reduces medication discrepancies [24] and supports interdisciplinary approaches such as perioperative optimization of senior health programs to improve postoperative outcomes [23].

Third, although no conclusion can be drawn from the non‐significant change observed in the pain‐assessment domain, the small number of items and the known complexity of pain assessment in older adults suggest that this competency may warrant dedicated attention in future educational work. Simulation‐based education and structured feedback on clinical cases could be explored as potential approaches to support pain‐assessment reasoning in older adults [25].

### 4.5 Methodological Considerations and Limitations

This study had several limitations. First, the single‐center design and small sample size limit the generalizability to other settings. The distribution of clinical experience may not reflect that of other institutions, thus affecting the observed educational impact.

Second, the use of an identical test for pre‐ and post‐intervention assessments is a methodological limitation. This may have introduced testing effects through increased familiarity, potentially confounding the true effects of the intervention. While consistent with SCT methodology [12], we cannot exclude test–retest effects. Differential improvements across domains provide evidence against pure test familiarity, as this would produce uniform improvements. However, the relative contribution of learning to familiarity remains unclear. Future studies should consider parallel SCT forms or control groups to better isolate the training effects.

Third, the one‐month follow‐up assessed only immediate knowledge retention, not lasting practice changes or patient outcomes.

Fourth, the uneven distribution of vignettes across domains may have affected their ability to detect changes in pain assessment competencies. The high variability and limited statistical power in the pain assessment domain make it difficult to draw definitive conclusions. This imbalance resulted from expert consensus on clinical importance and validated scenario availability.

In addition, SCT has inherent limitations when used as an assessment tool. Although it measures clinical reasoning in uncertain contexts, it does not directly measure clinical performance or prescribing behavior; the scores reflect concordance with expert opinion rather than an external guideline‐based standard. The pre‐intervention internal consistency (Cronbach's α of 0.542) raises reliability questions, although this improved post‐intervention (0.704) and aligns with other SCT validation studies [7, 8].

### 4.6 Future Directions

Future research should prioritize multicenter randomized controlled trials with larger sample sizes. Follow‐up assessments at 3, 6, and 12 months would determine optimal refresher training intervals. Studies should include control groups receiving standard education to establish inter‐professional collaborative training value. In particular, a controlled implementation trial could randomize centers or practitioners according to the interprofessional education program or usual education and assess whether the observed change in clinical reasoning translates into measurable differences in prescribing habits and in patient‐relevant outcomes (e.g., postoperative delirium, opioid‐related adverse events). Studies linking clinical reasoning to patient outcomes such as analgesic satisfaction and postoperative delirium are relevant to practice. Establishing connections between provider competence and patient outcomes validates educational interventions in anesthesiology [26]. Training models adapted to anesthesiologist time constraints warrant study. High‐fidelity simulation, reflective practice‐based learning, and online platforms enhance geriatric pain management competencies for an aging population with complex pain management issues [27].

## 5 Conclusion

This study suggests that collaborative geriatrician‐pharmacist training is associated with improved anesthesiologists' clinical reasoning regarding perioperative analgesic management in older adults. The significant improvement in SCT scores suggests this approach enhances concordance with expert opinion. Although our findings support interprofessional education, definitive conclusions about long‐term practice changes require larger longitudinal studies. The differential effects across domains, with gains in analgesic prescription but no significant change in pain assessment, highlight the complexity of clinical reasoning in geriatric anesthesiology. These findings suggest different aspects of geriatric pain management may require distinct educational approaches. As the population ages and the number of surgeries in older adults increases, optimizing perioperative pain management remains crucial. Our findings support further investigation of interprofessional education in anesthesiology training to enhance clinical reasoning in the care of older surgical patients.

## Author Contributions

Gilles Loggia: Study conceptualization and design; development and validation of the Script Concordance Test instrument; delivery of the collaborative educational intervention (geriatrician component); acquisition of study data; statistical analysis; drafting of the initial manuscript; critical revision of subsequent drafts; final approval of submitted version. Alexandre Meurant: Study conceptualization and design; delivery of the collaborative educational intervention (clinical pharmacist component); critical revision of the manuscript for important pharmaceutical expertise content; final approval of submitted version. Vincent Bonnet: Study conceptualization and design; statistical analysis and interpretation; critical revision of the manuscript for important methodological and anesthesiology expertise content; final approval of submitted version. Clément Gakuba: Critical revision of the manuscript for important anesthesiology expertise content; final approval of submitted version. Cédric Villain: Review and validation of the Script Concordance Test instrument; critical revision of the manuscript for important content related to pain management and geriatric medicine expertise; final approval of submitted version.

## Funding

The authors have nothing to report.

## Ethics Statement

This professional practice evaluation study was conducted in accordance with French regulatory requirements. The local Ethics Committee determined that formal ethics approval was not required as this educational research involved healthcare professionals rather than patients, with no patient data collection or modification to patient care protocols. All participating anesthesiologists provided informed consent. Data were anonymized and managed according to French data protection law and European GDPR regulations.

## Consent

Patient consent statement is not applicable for this study. This research involved healthcare professionals as participants, not patients. No patient data were collected or used in this study.

## Conflicts of Interest

The authors declare no conflicts of interest.

## Supporting information


**Figure S1:** Example of a Script Concordance Test (SCT) vignette with scoring methodology. This figure illustrates a complete SCT clinical vignette used to assess clinical reasoning in the context of perioperative pain management in older adult patients. The top panel presents a clinical scenario describing a 90‐year‐old post‐surgical patient with confusion. The middle panel shows the test item structure with the proposed therapeutic option (sustained‐release tramadol), new clinical information (reduced confusion), and a 5‐point Likert scale for the response. The bottom panels display the rating scale interpretation and aggregate scoring method based on the expert panel responses (*n* = 13).

**Figure S2:** Flow diagram of participant enrollment, allocation, and analysis. Flow of anesthesiologists (*n* = 19) through a prospective pre‐post educational intervention study (May–October 2023). Seventeen participants (89.5%) completed both pre‐ and post‐training Script Concordance Test assessments and were included in the primary analysis. Two participants (10.5%) were lost to follow‐up. Subgroup analyses were conducted by clinical experience level. SCT = Script Concordance Test.

**Data S1:** Script Concordance Test Instrument—Complete 20‐item instrument with clinical vignettes and scoring methodology.

**Data S2:** Educational Intervention Materials—Analgesic Treatment in Older Adults: Assessment and Treatment Specificities (Training Duration: 60 min).

## Data Availability

The data that support the findings of this study are available from the corresponding author upon reasonable request. The full Script Concordance Test instrument is available in Supporting Information S1.
