# Supplementary material for: Pain Management in Older Adults: An Interprofessional Training Approach Assessed by Script Concordance Tests
Source: Pain Pract. 2026 Oct 3;26(8):e70208. doi: 10.1111/papr.70208 (PMC13633571; doi:10.1111/papr.70208)
Supplement: Supplementary file 1 [file PAPR-26-0-s003.docx]

Supplementary Material 1

**Script Concordance Test Instrument**

*Complete 20-item instrument with clinical vignettes*

# Overview

This supplementary material presents the complete Script Concordance Test (SCT) instrument used in this study. The instrument comprises 20 clinical vignettes distributed across three domains:

**• Pain Assessment:** 2 items (vignettes 1, 13)

**• Analgesic Prescription:** 7 items (vignettes 2, 4, 6, 8, 9, 10, 11)

**• Adverse Effect Management:** 11 items (vignettes 3, 5, 7, 12, 14, 15, 16, 17, 18, 19, 20)

## Scoring methodology

Responses were scored using the aggregate scoring method based on a panel of 13 geriatricians. Full credit (1.0) was assigned to the modal expert response, with partial credit proportional to expert frequency for other responses.

# Domain 1: Pain Assessment (n=2)

**Vignette 1** *[Pain Assessment]*

**Clinical scenario:** A 75-year-old patient underwent total hip arthroplasty 24 hours ago. He experienced pain at the operated hip during the night. You plan to reassess him the next day.

| **If you were thinking of...** | **And you learn that...** | **Your assessment is now...** |
| --- | --- | --- |
| Assess pain using the Verbal Rating Scale (VRS/EVS) | The patient has Alzheimer's disease as the main medical history | -2 -1 0 +1 +2 |

**Scale:** *-2: Scale absolutely inappropriate; -1: Scale less justified; 0: No change; +1: Scale more justified; +2: Scale essential*

**Vignette 13** *[Pain Assessment]*

**Clinical scenario:** This same patient (Mme M, 78 years old, post hip replacement) is still hospitalized and remains on paracetamol and morphine. She still reports pain.

| **If you were thinking of...** | **And you learn that...** | **Your assessment is now...** |
| --- | --- | --- |
| Assess pain using the Numeric Rating Scale (NRS/EN) | The patient reports diffuse pain, not only at the operated hip | -2 -1 0 +1 +2 |

**Scale:** *-2: Scale absolutely inappropriate; -1: Scale less justified; 0: No change; +1: Scale more justified; +2: Scale essential*

# Domain 2: Analgesic Prescription (n=7)

**Vignette 2** *[Analgesic Prescription]*

**Clinical scenario:** For the same 75-year-old patient who underwent total hip arthroplasty 24 hours ago, you are considering prescribing an analgesic.

| **If you were thinking of...** | **And you learn that...** | **Your assessment is now...** |
| --- | --- | --- |
| Prescribe ibuprofen every 6 hours | He has a GFR of 77 mL/min | -2 -1 0 +1 +2 |

**Scale:** *-2: Absolutely contraindicated; -1: Less justified; 0: No change; +1: More justified; +2: Essential*

**Vignette 4** *[Analgesic Prescription]*

**Clinical scenario:** A 75-year-old patient is admitted for planned shoulder surgery following a fall. He also has hypertension treated with Ramipril and Amlodipine.

| **If you were thinking of...** | **And you learn that...** | **Your assessment is now...** |
| --- | --- | --- |
| Prescribe NSAIDs | The patient has a history of gastrointestinal ulcers | -2 -1 0 +1 +2 |

**Scale:** *-2: Absolutely contraindicated; -1: Less justified; 0: No change; +1: More justified; +2: Essential*

**Vignette 6** *[Analgesic Prescription]*

**Clinical scenario:** Mrs. L, 82 years old, is hospitalized for lumbar spinal stenosis surgery. In addition to postoperative pain, she also has pain characterized by burning sensations, tingling, painful cold, and electric shocks.

| **If you were thinking of...** | **And you learn that...** | **Your assessment is now...** |
| --- | --- | --- |
| Prescribe treatment for neuropathic pain | The resident prescribed gabapentin at an initial dose of 300 mg/day | -2 -1 0 +1 +2 |

**Scale:** *-2: Absolutely contraindicated; -1: Less justified; 0: No change; +1: More justified; +2: Essential*

**Vignette 8** *[Analgesic Prescription]*

**Clinical scenario:** A resident asks you to validate prescriptions for a 75-year-old patient. He tells you he hesitates and would like to prescribe another analgesic besides paracetamol because pain persists.

| **If you were thinking of...** | **And you learn that...** | **Your assessment is now...** |
| --- | --- | --- |
| Prescribe Lamaline (paracetamol + opium + caffeine) | The patient is hospitalized for intracranial hypertension | -2 -1 0 +1 +2 |

**Scale:** *-2: Absolutely contraindicated; -1: Less justified; 0: No change; +1: More justified; +2: Essential*

**Vignette 9** *[Analgesic Prescription]*

**Clinical scenario:** Mrs. X, 72 years old, underwent gallbladder surgery three days ago. She has moderate pain at the surgical scar. You must decide if NSAID administration would be appropriate.

| **If you were thinking of...** | **And you learn that...** | **Your assessment is now...** |
| --- | --- | --- |
| Prescribe ketoprofen | The patient has a blood pressure of 139/75 mmHg | -2 -1 0 +1 +2 |

**Scale:** *-2: Absolutely contraindicated; -1: Less justified; 0: No change; +1: More justified; +2: Essential*

**Vignette 10** *[Analgesic Prescription]*

**Clinical scenario:** Mr. D, 75 years old, underwent knee replacement surgery two days ago for advanced osteoarthritis. He was taking NSAIDs long-term for pain, stopped since surgery. He has moderate knee pain. You must decide if NSAID administration would be appropriate.

| **If you were thinking of...** | **And you learn that...** | **Your assessment is now...** |
| --- | --- | --- |
| Prescribe ketoprofen | The patient has a GFR of 45 mL/min | -2 -1 0 +1 +2 |

**Scale:** *-2: Absolutely contraindicated; -1: Less justified; 0: No change; +1: More justified; +2: Essential*

**Vignette 11** *[Analgesic Prescription]*

**Clinical scenario:** Mr. D, 75 years old, underwent knee replacement surgery two days ago. He was taking NSAIDs long-term, stopped since surgery. He has moderate knee pain. You must decide if NSAID administration would be appropriate.

| **If you were thinking of...** | **And you learn that...** | **Your assessment is now...** |
| --- | --- | --- |
| Prescribe ketoprofen | The patient has a history of stroke | -2 -1 0 +1 +2 |

**Scale:** *-2: Absolutely contraindicated; -1: Less justified; 0: No change; +1: More justified; +2: Essential*

# Domain 3: Adverse Effect Management (n=11)

**Vignette 3** *[Adverse Effect Management]*

**Clinical scenario:** A 78-year-old patient is admitted for hip surgery following a fracture. He also has mild to moderate dementia, for which he is on Haldol for behavioral symptom management. He also has COPD and benign prostatic hyperplasia with prescribed oxybutynin and Atrovent respectively.

| **If you were thinking of...** | **And you learn that...** | **Your assessment is now...** |
| --- | --- | --- |
| Prescribe tramadol 50 mg every 6 hours | A confused patient | -2 -1 0 +1 +2 |

**Scale:** *-2: Absolutely contraindicated; -1: Less justified; 0: No change; +1: More justified; +2: Essential*

**Vignette 5** *[Adverse Effect Management]*

**Clinical scenario:** A 72-year-old patient is admitted for non-urgent hip surgery. Clinical examination shows no obvious abnormalities. Laboratory tests are unremarkable except for mild chronic renal insufficiency. His usual treatments include Paracetamol, Macrogol, and Oxazepam.

| **If you were thinking of...** | **And you learn that...** | **Your assessment is now...** |
| --- | --- | --- |
| Prescribe Acupan (nefopam) 20 mg every 4 hours as needed for postoperative pain | The patient has a history of Alzheimer's disease | -2 -1 0 +1 +2 |

**Scale:** *-2: Absolutely contraindicated; -1: Less justified; 0: No change; +1: More justified; +2: Essential*

**Vignette 7** *[Adverse Effect Management]*

**Clinical scenario:** A 90-year-old patient operated for right femoral neck fracture has postoperative confusion with agitation and aggression. She wants to get up while weight-bearing is prohibited. Her history includes hypertension and mild Alzheimer's dementia. Examination finds positive Algoplus. She is currently on paracetamol 1g x3/day and Acupan 20mg every 4 hours.

| **If you were thinking of...** | **And you learn that...** | **Your assessment is now...** |
| --- | --- | --- |
| Prescribe tramadol LP 100 mg x2/day and stop Acupan | The patient seems less confused than yesterday | -2 -1 0 +1 +2 |

**Scale:** *-2: Absolutely contraindicated; -1: Less justified; 0: No change; +1: More justified; +2: Essential*

**Vignette 12** *[Adverse Effect Management]*

**Clinical scenario:** Mrs. M, 78 years old, had hip replacement surgery following a fall 3 days ago. Postoperatively she was prescribed paracetamol and morphine. She still has moderate pain. You must decide if NSAID administration would be appropriate.

| **If you were thinking of...** | **And you learn that...** | **Your assessment is now...** |
| --- | --- | --- |
| Prescribe ketoprofen | The patient shows decreased pain | -2 -1 0 +1 +2 |

**Scale:** *-2: Absolutely contraindicated; -1: Less justified; 0: No change; +1: More justified; +2: Essential*

**Vignette 14** *[Adverse Effect Management]*

**Clinical scenario:** Mrs. V, 76 years old, underwent major abdominal surgery for intestinal tumor resection three days ago. She has significant pain at the surgical incision despite standard analgesic treatment with paracetamol.

| **If you were thinking of...** | **And you learn that...** | **Your assessment is now...** |
| --- | --- | --- |
| Prescribe Acupan (nefopam) | The patient had sudden brief loss of consciousness with sudden generalized muscle contractions | -2 -1 0 +1 +2 |

**Scale:** *-2: Absolutely contraindicated; -1: Less justified; 0: No change; +1: More justified; +2: Essential*

**Vignette 15** *[Adverse Effect Management]*

**Clinical scenario:** Mrs. Z, 68 years old, overweight, with history of COPD, peripheral arterial disease, hiatal hernia, underwent recent hernioplasty. She has moderate pain at the operated area despite standard paracetamol treatment at 4g/day.

| **If you were thinking of...** | **And you learn that...** | **Your assessment is now...** |
| --- | --- | --- |
| Prescribe Acupan (nefopam) | Decreased respiratory rate on clinical examination | -2 -1 0 +1 +2 |

**Scale:** *-2: Absolutely contraindicated; -1: Less justified; 0: No change; +1: More justified; +2: Essential*

**Vignette 16** *[Adverse Effect Management]*

**Clinical scenario:** Mr. B, 83 years old, underwent surgery for femoral neck fracture. At day 2 post-operation, he can weight-bear but remains painful despite the prescribed level I analgesic. You are considering prescribing a level II analgesic.

| **If you were thinking of...** | **And you learn that...** | **Your assessment is now...** |
| --- | --- | --- |
| Prescribe tramadol | The department's tramadol prescription protocols systematically use a minimum dose of 200 mg/day | -2 -1 0 +1 +2 |

**Scale:** *-2: Absolutely contraindicated; -1: Less justified; 0: No change; +1: More justified; +2: Essential*

**Vignette 17** *[Adverse Effect Management]*

**Clinical scenario:** This same patient (Mr. B) has constipation following abdominal surgery and analgesic prescriptions.

| **If you were thinking of...** | **And you learn that...** | **Your assessment is now...** |
| --- | --- | --- |
| Prescribe laxatives | Bowel function is progressively resuming despite being on morphine | -2 -1 0 +1 +2 |

**Scale:** *-2: Absolutely contraindicated; -1: Less justified; 0: No change; +1: More justified; +2: Essential*

**Vignette 18** *[Adverse Effect Management]*

**Clinical scenario:** Mr. J, 84 years old, is being treated for renal stone extraction. To relieve his intense pain he receives level I then II analgesics. He has an EVS of 4 and is becoming confused.

| **If you were thinking of...** | **And you learn that...** | **Your assessment is now...** |
| --- | --- | --- |
| Prescribe high-dose morphine | He has a history of Alzheimer's disease | -2 -1 0 +1 +2 |

**Scale:** *-2: Absolutely contraindicated; -1: Less justified; 0: No change; +1: More justified; +2: Essential*

**Vignette 19** *[Adverse Effect Management]*

**Clinical scenario:** Mr. T, 79 years old, lives in a nursing home since his stroke where he has decreased autonomy and swallowing disorders with aspiration. He underwent left wrist osteosynthesis surgery due to recurrent falls related to his stroke. Since surgery, he takes paracetamol and tramadol but oral intake is difficult due to swallowing disorders.

| **If you were thinking of...** | **And you learn that...** | **Your assessment is now...** |
| --- | --- | --- |
| Prescribe fentanyl given difficult oral intake | He has moderate pain | -2 -1 0 +1 +2 |

**Scale:** *-2: Absolutely contraindicated; -1: Less justified; 0: No change; +1: More justified; +2: Essential*

**Vignette 20** *[Adverse Effect Management]*

**Clinical scenario:** A 77-year-old patient is admitted for planned herniated disc surgery. Your department's postoperative pain management protocols allow systematic prescription of paracetamol, morphine, and one dose of NSAID.

| **If you were thinking of...** | **And you learn that...** | **Your assessment is now...** |
| --- | --- | --- |
| Prescribe the NSAID | The patient has a history of atrial fibrillation for which he takes vitamin K antagonists | -2 -1 0 +1 +2 |

**Scale:** *-2: Absolutely contraindicated; -1: Less justified; 0: No change; +1: More justified; +2: Essential*

# Expert Panel Information

The scoring criteria were established by a panel of 13 geriatricians with expertise in analgesic prescriptions for older adults. Panel members were recruited from academic and hospital settings in France.

**Panel characteristics:**

• Number of experts: 13

• Specialty: Geriatric medicine

• Setting: Academic and hospital geriatricians

• Expertise: Analgesic prescription in older adults

Scoring followed the aggregate method described by Fournier et al. (BMC Med Inform Decis Mak 2008;8:18), where full credit (1.0) is assigned to modal expert responses, with partial credit proportional to expert frequency for other responses.
