# Supplementary material for: Pain Management in Older Adults: An Interprofessional Training Approach Assessed by Script Concordance Tests
Source: Pain Pract. 2026 Oct 3;26(8):e70208. doi: 10.1111/papr.70208 (PMC13633571; doi:10.1111/papr.70208)
Supplement: Supplementary file 2 [file PAPR-26-0-s004.docx]

Supplementary Material 2

**Educational Intervention Materials**

Analgesic Treatment in Older Adults:

Assessment and Treatment Specificities

*Training Duration: 60 minutes*

# Contents

Part 1: Pain Assessment in Older Adults

1.1 Self-assessment scales

1.2 Hetero-assessment scales

1.3 Clinical algorithm for scale selection

Part 2: Potentially Inappropriate Analgesics in Older Adults

2.1 Paracetamol (Acetaminophen)

2.2 Non-Steroidal Anti-Inflammatory Drugs (NSAIDs)

2.3 Nefopam

2.4 Weak Opioids

2.5 Strong Opioids

Part 3: Postoperative Pain Protocols

Part 4: Neuropathic Pain Management

References

# Part 1: Pain Assessment in Older Adults

This section covers validated pain assessment tools for older adults, based on recommendations from the French National Authority for Health (HAS) and professional societies (SFETD, SFGG, SFAP).

## 1.1 Self-Assessment Scales

**Key principles:**

- Numeric Rating Scale (NRS/EN) or Verbal Rating Scale (VRS/EVS) are preferred
- **Visual Analogue Scale (VAS) is NOT recommended** for older adults due to cognitive demands
- Self-assessment is reliable for patients with mild-moderate cognitive impairment (MMSE > 18/30) - expert consensus
- Always attempt self-assessment first, even in patients with verbal communication difficulties

**Verbal Rating Scale (VRS/EVS) - Recommended**

| **Score** | **Description** |
| --- | --- |
| 0 | No pain |
| 1 | Mild pain |
| 2 | Moderate pain |
| 3 | Intense pain |
| 4 | Extremely intense pain |

## 1.2 Hetero-Assessment Scales

When self-assessment is unreliable or impossible, use observational scales:

**ALGOPLUS Scale - Acute Pain Detection**

- 5 items scored Yes/No
- **Threshold score: ≥2/5 indicates pain**
- Initially validated for acute pain, but can be used for most pain screening

| **Item** | **Observation** |
| --- | --- |
| **1. Face** | Frowning, grimacing, clenched jaw, frozen face |
| **2. Gaze** | Inattentive, fixed, distant or pleading look, tears, closed eyes |
| **3. Complaints** | "Ouch", "It hurts", moaning, crying |
| **4. Body** | Withdrawal, protection of area, refusal to mobilize, frozen postures |
| **5. Behavior** | Agitation, aggression, clinging |

**DOLOPLUS Scale - Chronic Pain Assessment**

- 10 items scored 0-3
- **Threshold score: ≥5/30 indicates pain**
- Primarily for chronic pain detection
- **Limitation:** Requires knowledge of patient's baseline state - limits use in acute care settings

**ECPA Scale (Behavioral Pain Scale for Elderly)**

- 8 items scored 0-4
- **No threshold score** - used for monitoring
- Dedicated to chronic and recurrent pain during care

## 1.3 Clinical Algorithm for Pain Assessment Scale Selection

*Based on Bonin-Guillaume S & Rat P. OBM Geriatrics 2020; Capriz et al. Revue de Gériatrie 2021*

**STEP 1: Can the patient self-assess pain?**

**IF YES →** Use self-assessment with VRS (5-point scale)

• Score ≥ 2 → TREAT

• Score < 2 → RE-EVALUATE

**IF NO →** Proceed to rapid hetero-assessment with ALGOPLUS

**STEP 2: ALGOPLUS Results**

**Score = 0 →** Complete hetero-assessment (Doloplus, ECPA, PACSLAC-F)

**Score = 1 →** Complete hetero-assessment → Consider treatment?

**Score ≥ 2 → TREAT**

**ALWAYS RE-EVALUATE AFTER TREATMENT**

# Part 2: Potentially Inappropriate Analgesics in Older Adults

This section addresses medication-specific considerations based on REMEDIeS criteria (Roux et al. 2021) and STOPP/START v3 criteria (O'Mahony et al. 2023).

## 2.1 Paracetamol (Acetaminophen)

**✓ FIRST-LINE ANALGESIC - but requires dose adjustment in older adults**

**Dosing recommendations for patients ≥75 years OR ≥65 years with multiple comorbidities:**

(Due to decreased glutathione reserves)

| **Parameter** | **Maximum Dose** |
| --- | --- |
| Maximum daily dose | **3 g/day** |
| Maximum single dose | **20 mg/kg/dose** |
| Maximum daily dose (weight-based) | **60 mg/kg/day** |

**⚠ CAUTION:** Effervescent formulations contain significant NaCl - avoid in heart failure or hypertension

## 2.2 Non-Steroidal Anti-Inflammatory Drugs (NSAIDs)

**⚠ TO BE AVOIDED - Prescription of last resort only**

**Contraindications according to REMEDIeS and STOPP/START v3:**

| **REMEDIeS Criteria** | **STOPP/START v3 Criteria** |
| --- | --- |
| • Indomethacin (↑ neuropsychic effects)  • Chronic heart failure  • Chronic renal failure  • Peptic ulcers  • Oral anticoagulants  • Low-dose aspirin  • ACE inhibitors / ARBs  • Diuretics | • History of coronary/cerebral/peripheral vascular disease (↑ thrombosis risk)  • Heart failure requiring loop diuretics  • History of peptic ulcer or GI bleeding (unless with PPI)  • Concurrent VKA, DOACs  • eGFR < 50 mL/min/1.73m²  • Severe hypertension (>170/100 mmHg) |

*If NSAIDs absolutely necessary (SFAR 2019 recommendations):*

- Use with caution only if paracetamol ineffective
- Low doses, short duration
- Associate with PPI
- Prefer non-selective NSAIDs in patients with atherosclerosis

## 2.3 Nefopam (Acupan®)

**⚠ TO BE AVOIDED - Prescription of last resort only**

**Benefits:**

- Reduces opioid consumption and pain scores by 30-40%
- No respiratory depression

**Risks (anticholinergic properties):**

| **Peripheral Effects** | **Central Effects** |
| --- | --- |
| • Tachycardia  • Urinary retention  • Angle-closure glaucoma | • Hallucinations  • Confusion  • Seizures (↓ seizure threshold) |

**REMEDIeS:** "Nefopam: Anticholinergic and other adverse effects such as nausea and convulsions"

## 2.4 Weak Opioids (Codeine, Tramadol, Opium)

**✓ Have their place in acute and chronic pain treatment**

**Key principles:**

- Initiate at reduced dose
- **Tramadol is preferred for mixed pain** (nociceptive + neuropathic component)
- **Always co-prescribe laxatives**

**Tramadol Titration Protocol:**

| **CrCl (mL/min)** | **<45 kg** | **45-60 kg** | **>60 kg** |
| --- | --- | --- | --- |
| **≥30** | 5 drops x3/day | 7 drops x3/day | 10 drops x3/day |
| **<30** | 3 drops x3/day | 4 drops x3/day | 5 drops x3/day |

*Titration instructions:* "Three times daily" = 6-8 hours between doses. Adjust every 48-72h based on pain and side effects. Increase by 25-50% per dose.

**REMEDIeS: Tramadol > 200 mg/day**

• Prolonged elimination half-life in individuals >75 years

• CNS adverse effects: confusion, dizziness, falls risk

• Serotonergic properties → serotonin syndrome risk with other serotonergic drugs

**Cytochrome P450 2D6 Polymorphism:**

Codeine, tramadol, and oxycodone are metabolized via CYP2D6 to active metabolites:

| **Drug** | **Active Metabolite** | **Note** |
| --- | --- | --- |
| Codeine | Morphine | Codeine inactive without metabolism |
| Oxycodone | Oxymorphone | 14x more potent than parent |
| Tramadol | M1 metabolite | Active metabolite |

**Clinical implication:** 5-10% are ultra-rapid metabolizers (↑ toxicity risk); 15-25% are poor/intermediate metabolizers (↓ efficacy). For ~1/3 of patients, effects are unpredictable → **"Start low, go slow"**

## 2.5 Strong Opioids

**Recommended initial doses for opioid-naive older adults:**

| **Drug** | **Oral Route** | **SC/IV Route** |
| --- | --- | --- |
| **Morphine** | 15-30 mg/day (2.5-5 mg/dose q4-6h) | 5-10 mg/day (SC) or 7.5-15 mg/day (IV) |
| **Oxycodone** | 10-20 mg/day | 7-14 mg/day |

**Key recommendations:**

- Choose the lowest recommended dose for frail patients
- Prefer least invasive route; favor oral route when possible
- Introduce one analgesic at a time to evaluate efficacy and side effects
- Titration with immediate-release opioid q4-6h (or extended-release q12h if living situation requires)
- Consider transmucosal fentanyl for breakthrough pain in well-controlled patients
- **No maximum dose limitation due to age alone**

**Note:** Limited pharmacokinetic/pharmacodynamic data in older adults - no evidence to recommend one opioid over another. New orodispersible formulations (Actiskenan 1mg, 2.5mg) useful for patients with swallowing difficulties.

# Part 3: Postoperative Pain Protocols

*Institution-specific protocols for opioid-naive older adults (CHU de Caen)*

## 3.1 Minor Surgery (Digestive/Urological) - Oral Route

| **Medication** | **Protocol** |
| --- | --- |
| **Paracetamol** | Systematic: 1g x3/day PO (500mg orodispersible) |
| **Antispasmodic** | Systematic: Phloroglucinol 80mg x2, 3x/day PO |
| **Tramadol drops** | 5-5-5 drops + 5 as needed |
| **If tramadol ineffective:** | Switch to Actiskenan orodispersible 2.5mg: 1-1-1 + PRN (min interval 1h, max 4 tablets/4h) |
| **Laxatives** | Macrogol (Transipeg): 2-0-0 (except if stoma) |

## 3.2 Major Surgery (Digestive/Urological) - IV then Oral Relay

**Days 1-2: IV medications**

| **Medication** | **Protocol** |
| --- | --- |
| **Paracetamol IV** | Systematic: 1g x3/day IV for 2 days |
| **Antispasmodic IV** | 8 ampoules phloroglucinol in 1L glucose over 24h for 2 days |
| **Morphine IV** | 2-2-2-2 mg IV (or SC if no IV access) |
| **Laxatives** | Macrogol: 2-0-0 (except if stoma) |

**From Day 3: Systematic oral relay**

- Paracetamol: 1g x3/day PO (500mg orodispersible)
- Antispasmodic: Phloroglucinol 80mg x2, 3x/day PO
- Actiskenan orodispersible 2.5mg: 1-1-1 + PRN (min interval 1h, max 4 tablets/4h)

## 3.3 Required Monitoring

- **Sedation score and respiratory rate**
- **Pain scores:** VRS for communicating patients; Algoplus for non-communicating patients
- **Watch for:** Hypovigilance, agitation, incoherence → request geriatric consultation if present

# Part 4: Neuropathic Pain Management

*Based on: Moisset X et al. Douleur et Analgésie 2020*

## 4.1 Therapeutic Algorithm

| **Line** | **Localized Peripheral NP** | **Other Peripheral or Central NP** |
| --- | --- | --- |
| **1st Line** | • Lidocaine patches (1-3 patches, 12h/day)  • TENS (≥30 min/day) | • SNRI: Duloxetine (60-120 mg/day) or Venlafaxine (150-225 mg/day)  • Gabapentin (1200-3600 mg/day)  • Tricyclic antidepressants (10-150 mg/day) |
| **2nd Line** | • Capsaicin 8% patches (1-4 patches/3 months)  • Botulinum toxin A (50-300 units/3 months) | • Pregabalin (150-600 mg/day)  • Tramadol (100-400 mg/day)  • Combination therapy (antidepressant + gabapentinoid) |
| **3rd Line** | • rTMS of M1 (>5Hz, >1200 pulses/session)  • Spinal cord stimulation (FBSS, diabetic polyneuropathy)  • Strong opioids alone or in combination (last resort, <150 MME/day, after abuse risk assessment) | |

**Note:** Psychotherapy should be considered at all stages as adjunctive treatment

# References

1. Hadjistavropoulos T, et al. An interdisciplinary expert consensus statement on assessment of pain in older persons. Clin J Pain 2007;23(1 Suppl):S1-43. PMID: 17179836
2. Bonin-Guillaume S, Rat P. Assessment tools for pain in the elderly: what's new? OBM Geriatrics 2020;4(4):1-15.
3. Capriz F, et al. Évaluation de la douleur dans le grand âge: où en sommes-nous en 2021? Revue de Gériatrie 2021;46:205-212.
4. Roux B, et al. REview of potentially inappropriate MEDIcation pr[e]scribing in Seniors (REMEDIeS): French implicit and explicit criteria. Eur J Clin Pharmacol 2021;77:1713-1724. PMID: 34117909
5. O'Mahony D, et al. STOPP/START criteria for potentially inappropriate prescribing in older people: version 3. Eur Geriatr Med 2023;14:625-632. PMID: 37256475
6. American Geriatrics Society 2023 updated AGS Beers Criteria® for potentially inappropriate medication use in older adults. J Am Geriatr Soc 2023;71:2052-2081. PMID: 37139824
7. Société Française d'Anesthésie et de Réanimation. Prise en charge de la douleur postopératoire chez l'adulte et l'enfant. SFAR 2008 (mise à jour 2019).
8. Guirimand F, et al. The effects of nefopam on morphine consumption in postoperative patients. Pain 1999;81:153-159. PMID: 10353502
9. Durrieu G, et al. Pharmacovigilance and adverse drug reactions. Fundam Clin Pharmacol 2007;21:555-563.
10. Annequin D. Opioïdes et polymorphisme génétique: impact sur la prescription. Repères en Gériatrie 2023;25:18-22.
11. Moisset X, et al. Recommandations françaises pour la prise en charge de la douleur neuropathique. Douleur et Analgésie 2020;33:79-94.
12. Haute Autorité de Santé. Test de concordance de script (TCS). HAS 2017.
13. Centre de pédagogie appliquée aux sciences de la santé. Construire un test de concordance de script. Université de Montréal.
